# *O*-GlcNAcylation of fatty acid synthase is required for its proper subcellular localization, expression level, and activity

**DOI:** 10.1016/j.jbc.2025.110497

**Published:** 2025-07-18

**Authors:** Dimitri Vanauberg, Céline Schulz, Sadia Raab, Gabriela Fuentes-GarcÍa, Emilie Cadart, Quentin Lemaire, Stéphanie Olivier-Van Stichelen, Fabrice Bray, Guillaume Brysbaert, Yannick Rossez, Stéphan Hardivillé, Tony Lefebvre

**Affiliations:** 1 University of Lille, CNRS, UMR 8576 - UGSF - Unité de Glycobiologie Structurale et Fonctionnelle, Lille, France; 2 Medical College of Wisconsin, Department of Biochemistry, Wisconsin, Milwaukee, USA; 3 University of Lille, CNRS, UAR 3290-MSAP-Miniaturisation pour la Synthèse, l'Analyse et la Protéomique, Lille, France

**Keywords:** fatty acid synthase, fatty acids, *O*-GlcNAcylation, *O*-GlcNAc transferase, liver cancer cells

## Abstract

Fatty Acid Synthase (FASN) is involved in various fundamental cellular processes through its pivotal role in producing fatty acids through the *de novo* lipogenesis pathway. FASN is frequently overexpressed in tumors and participates in cancer cell proliferation. Little has been documented regarding post-translational modifications of FASN. We previously demonstrated that *O*-GlcNAcylation regulates FASN in mice livers and in the HepG2 hepatic cancer cell line. In the present study, we show that modulation of global *O-*GlcNAcylation levels impacts fatty acids production in HepG2 cells. We identified serine 595 and threonine 980 as major *O*-GlcNAcylation sites. While mutation of S595 moderately affects FASN behavior, T980 is crucial for FASN expression, membrane localization, homodimerization, stability, and activity in Hep3B cells. This residue is necessary for FASN properties, promoting cell survival, cell proliferation, and cell cycle progression. Our results suggest that targeting FASN at T980 may open an interesting path for controlling its catalytic activity.

Hepatic cancer is an emerging major public health concern, ranking as the sixth most common cancer worldwide and the third leading cause of cancer-related mortality (1). Hepatocellular carcinoma (HCC), the most represented subtype of hepatic cancer, is characterized by a dysregulation of lipid metabolism and storage, with an accumulation of fat in diseased hepatocytes (2). This phenomenon results in part from the overexpression of enzymes involved in the *de novo* lipogenesis pathway, including fatty acid synthase (FASN), whose expression is associated with a poor prognosis in HCC (3, 4).

FASN is a ubiquitous and high-molecular-weight enzyme (273 kDa) that functions as a homodimer (4, 5). The enzyme is mainly located in the cytosolic compartment but also interacts with lipid microdomains (6). FASN produces fatty acids using acetyl-CoA, malonyl-CoA and NAPDH,H^+^ *via* 7 different catalytic activities. Fatty acids can be stored following esterification in the form of lipid droplets for energy storage, be used for membranes building, protein palmitoylation or to generate second messengers (7). Therefore, FASN is involved in cell proliferation and contributes to many hallmarks of cancers (8). Often overlooked, FASN is regulated by post-translational modifications (PTM) among which *O*-β-N-acetyl-D-glucosaminylation or *O*-GlcNAcylation (9).

*O*-GlcNAcylation consists of the addition of a single N-acetyl-D-glucosamine (GlcNAc) onto the hydroxyl group of serine and threonine residues of thousands of proteins by *O*-GlcNAc transferase (OGT) (10, 11). Like phosphorylation, this modification is dynamic, as *O*-GlcNAcase (OGA) removes the GlcNAc group from proteins. *O*-GlcNAcylation is a nutrient-dependent modification since the level of the nucleotide-sugar UDP-GlcNAc, the donor of the GlcNAc group, depends on the metabolism of glucose, fatty acids, amino acids, and nucleotides (11). Like FASN, OGT is overexpressed in cancers, including HCC, and is thought to participate in many hallmarks of cancer by regulating protein expression, activity, subcellular location, and interactions (12).

Due to their respective pro-oncogenic properties, the investigation of the relationship between FASN and OGT in HCC is of particular interest. First, *O-*GlcNAcylation enhances lipid accumulation in HepG2 hepatic cancer cells (13). Furthermore, we demonstrated that FASN and OGT physically interact in the liver (9). Indeed, *O*-GlcNAcylation of FASN, in mice livers and in HepG2 cells, promotes its interaction with the ubiquitinase Ubiquitin Specific Peptidase 2a (USP2a). This results in reduced FASN ubiquitination, decreasing its proteasomal degradation and, therefore, increasing its activity. In mouse hepatocytes, *O*-GlcNAcylation also promotes FASN expression by modifying the carbohydrate-responsive element-binding protein (ChREBP) transcription factor (14). ChREBP *O*-GlcNAcylation increases its stability and its transcriptional activity towards *FASN*. In addition, FASN inhibition reduces OGT protein amount and conversely OGT inhibition decreases FASN content, disturbing cell cycle progression and reducing cancer cell viability (15). FASN can also inhibit OGA in U2OS cells under oxidative stress to promote cancer cell survival (16). Finally, FASN *O*-GlcNAcylation promotes HeLa cell survival during starvation (17). Altogether, these data highlight the complex relationship between FASN and *O*-GlcNAcylation in cancer cells.

By combining fatty acid analyses, mass spectrometry-based *O*-GlcNAc site-mapping, expression of FASN *O*-GlcNAcylation mutants, Western blotting, and cellular assays, this study highlights the role of *O*-GlcNAcylation on FASN in HCC cells. We first showed that *O*-GlcNAcylation regulates fatty acid synthesis in HepG2 cells. We then identified serine 595 (S595) and threonine 980 (T980) of FASN as *O*-GlcNAcylation sites. Site-directed mutagenesis showed that T980 is essential for FASN expression level, homodimerization, membrane residence, stability, and enzymatic activity in Hep3B cells. Furthermore, T980 sustains FASN activity, therefore promoting Hep3B cells' survival and proliferation as cell cycle progression.

## Results

### Fatty acid synthesis is regulated by O-GlcNAcylation levels

**Figure 1:**
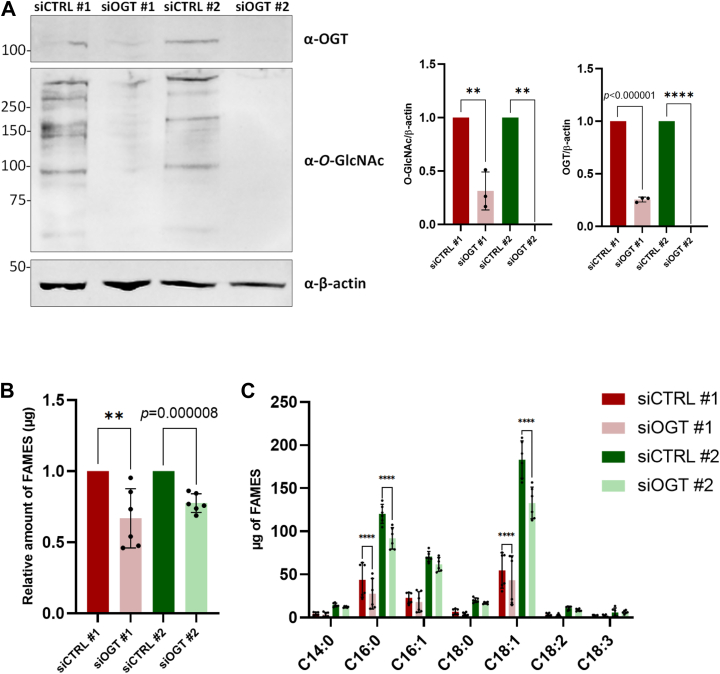
**OGT knockdown negatively impacts fatty acids synthesis**. HepG2 cells were transfected with two different sets of siRNA targeting OGT mRNA. Cell lysates were analyzed by Western blot (n = 3 for siOGT#1 and n = 6 for siOGT#2) according to their OGT and *O*-GlcNAc contents (*A*). Molecular mass markers are indicated on the left (kDa). Optical densities were measured and normalized with β-actin expression. Global (*B*) and individual (*C*) amounts of FAMES from HepG2 transfected cells were measured by GC-FID (n = 6). Data are presented with means ± SD. ∗∗*p* < 0.01; ∗∗∗∗*p* < 0.0001.

**Figure 2:**
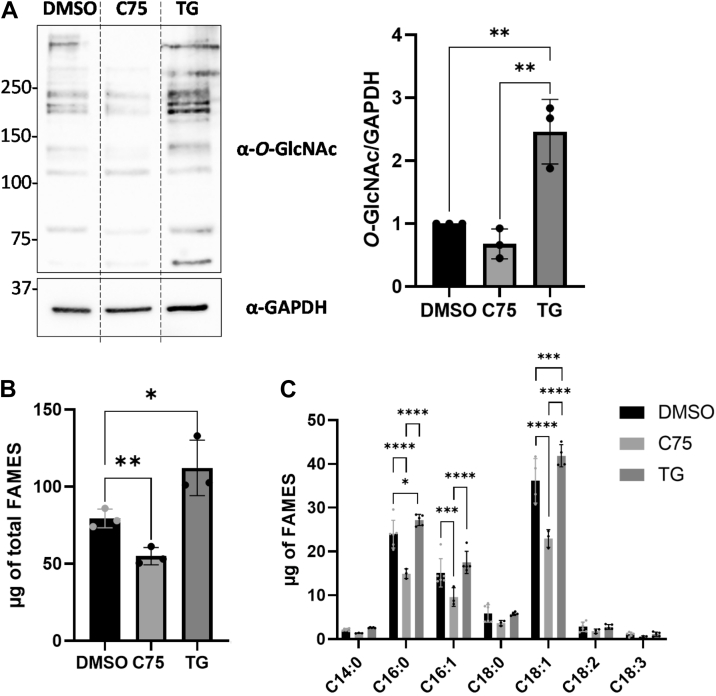
**OGA inhibition positively impacts fatty acids synthesis**. Cell lysates from HepG2 treated with the FASN inhibitor C75 or OGA inhibitor Thiamet-G (TG) were analyzed by Western blot (n = 3) according to their *O*-GlcNAc content **(***A*). Optical densities were measured and normalized with GAPDH expression. Global (*B*) and individual (*C*) amounts of FAMES from HepG2-treated cells were measured by GC-FID (n = 6). Data are presented with means ± SD. ∗*p* < 0.05; ∗∗*p* < 0.01; ∗∗∗*p* < 0.001; ∗∗∗∗*p* < 0.0001.

To expand on our previous data indicating a positive cross-regulation between FASN and OGT (9, 15), we performed fatty acid analyses on HepG2 cells (Figs. 1 and 2). First, *OGT* was silenced by incubating two different sets of siRNA on HepG2 cells for 96 h, resulting in a significant reduction of both OGT and *O*-GlcNAcylation levels (Fig. 1*A*). The measurement of global fatty acid methyl esters (FAMES) by GC-FID indicated that down-regulating *O*-GlcNAcylation significantly decreased fatty acids production (Fig. 1*B*), particularly palmitic acid (C16:0) - the primary and final product of FASN - and oleic acid (C18:1) (Fig. 1*C*). Conversely, increased*-O*-GlcNAcylation level by inhibiting OGA with Thiamet-G (TG) (Fig. 2*A*) significantly increased global FAMES (Fig. 2*B*), and more particularly palmitic acid (C16:0), palmitoleic acid (C16:1), and oleic acid (C18:1) (Fig. 2*C*). Silencing of *OGA* by siRNA transfection also significantly increased global FAMES (Fig. S1). In contrast, C75, an inhibitor of FASN, significantly reduced global FAMES, palmitic acid (C16:0), palmitoleic acid (C16:1), and oleic acid (C18:1). Taken together, these experiments demonstrate that fatty acid production is positively regulated by *O*-GlcNAcylation in hepatic cancer cells.

### FASN is O-GlcNAcylated at S595 and T980, two sites conserved along evolution

To map FASN *O*-GlcNAcylation sites, LC-MS/MS analyses were performed on the enzyme immunopurified from HepG2 protein lysate. Mass spectrometry revealed two major sites of *O*-GlcNAcylation at threonine 980 (Fig. S2*A*) and serine 595 (Fig. S2*B*). Of particular interest, *O*-GlcNAcylation at T980 was previously reported by Woo and collaborators in 2018 (18).

### Mutation of FASN at T980 O-GlcNAcylation site impairs protein level, membrane localization, dimerization, and stability of the enzyme

**Figure 3:**
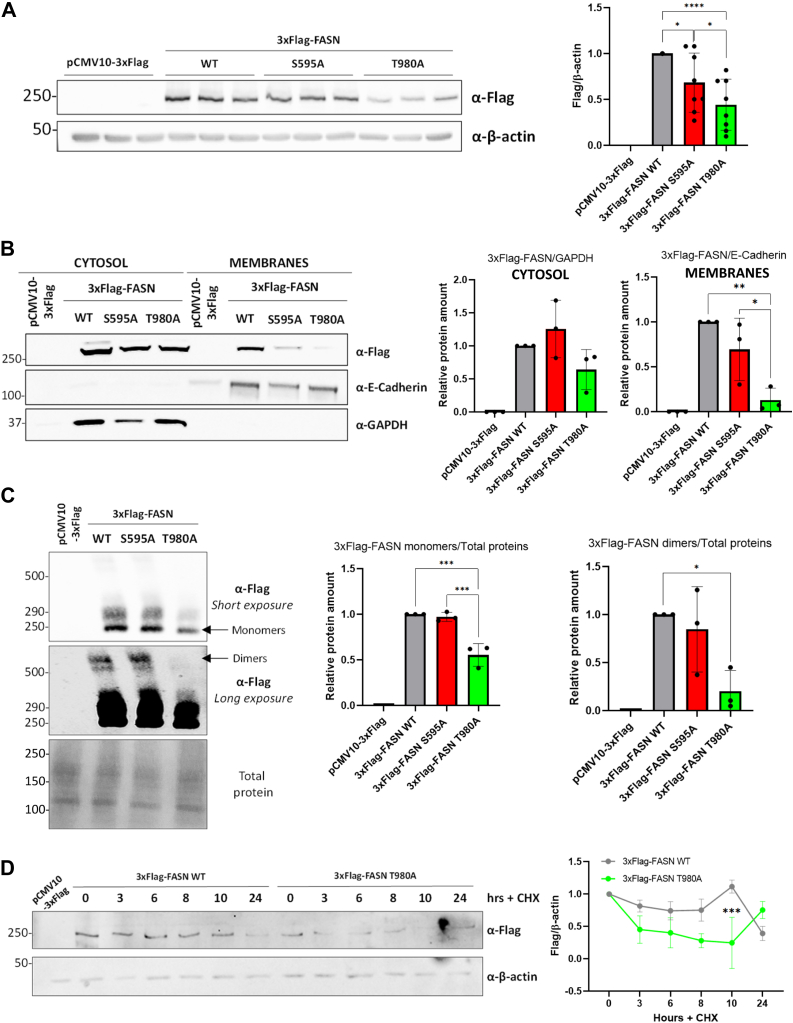
**Mutation of the T980 *O*-GlcNAcylation site reduces FASN expression, membrane association, homodimerization, and stability**. Hep3B cells were transfected with an empty vector or 3xFlag-FASN (wild type and mutants) expressing vectors. Cell lysates were analyzed by Western blot (n = 8) by using an anti-Flag antibody (*A*). Molecular mass markers are indicated on the left (kDa). Optical densities were measured and normalized with β-actin expression. *B*, cytosolic and membrane-associated 3xFlag-FASN contents were prepared by cell fractionation and analyzed by Western blot (n = 3). Fractionation efficiency was assessed using anti-GAPDH and anti-E-Cadherin antibodies for cytosolic and membrane fractions, respectively. Optical densities were measured and normalized with these markers. *C*, cell lysates were analyzed by Native-PAGE (n = 3) according to their 3xFlag-FASN monomers (∼250 kDa) and dimers (∼500 kDa) contents. Optical densities were measured and normalized with total protein. *D*, CHX chase assay was performed by treating cells with 40 μg/ml CHX for the indicated time periods (0–24 h). Data are presented with means ± SD. *∗p < 0.05;* ∗∗*p* < 0.01; *∗∗∗p* < 0.001; *∗∗∗∗p* < 0.0001.

To investigate the impact of S595 and T980 *O*-GlcNAcylation sites on FASN regulation, the 3xFlag-FASN S595A and T980A mutants were constructed and expressed in Hep3B cells in parallel to empty vector (negative control) (Fig. 3). The glycosylation status of the different forms of FASN and their ability to interact with OGT were evaluated respectively by enrichment onto WGA-coupled beads (Fig. S3*A*) and by co-immunoprecipitation experiments (Fig. S3*B*). The results seem to indicate that T980 is a major *O*-GlcNAcylation site but that mutation of FASN does not affect its interaction with OGT. A decrease in FASN level was observed for the T980A isoform whereas only a slight decrease was noticed for the S595A isoform compared to wild type FASN (Fig. 3*A*). While FASN is mainly localized in the cytosolic compartment, a fraction of the enzyme is resident of membranes (6). Therefore, the distribution of the mutants *versus* the wild-type isoforms was evaluated by cell fractionation (Fig. 3*B*). As a result, FASN T980A was significantly less located in the membrane fraction compared to FASN wild type and FASN S595A isoforms. The efficiency of cell fractionation was assessed by using antibodies directed against GAPDH and E-cadherin (respectively markers of cytosolic and membranes fractions). FASN is essentially active as a homodimer (4, 5, 7). Then, we performed native-PAGE to examine the impact of both mutations on the dimerization of the enzyme (Fig. 3*C*). Native-PAGE allowed the identification of the monomeric (∼250 kDa) and dimeric (∼500 kDa) forms of the enzyme (Fig. 3*C*). Of particular interest, a significant decrease in both forms was observed for FASN T980A but with an even more significant reduction for dimeric forms than monomers. Regarding the impact of mutation of FASN at T980, we finally wondered whether this site could be involved in protein stability. For that purpose, we used cycloheximide (CHX), an inhibitor of protein synthesis (Fig. 3*D*). While we observe a slight decrease in the wild-type version of FASN along the time-course experiment, the T980A mutant form decreases much more rapidly, from 3 h after the application of CHX, indicating that the T980 site plays a preponderant role in the expression of the enzyme. We have in a last time used MG132, a proteasome inhibitor, to test its capability to reduce the degradation of FASN mutants, which was partly observed (Fig. S4). Thus, the mutation of FASN at T980 suggests that *O*-GlcNAcylation of this residue is key for regulating the properties of the enzyme.

### Mutation of FASN at T980 reduces lipid droplets number

**Figure 4:**
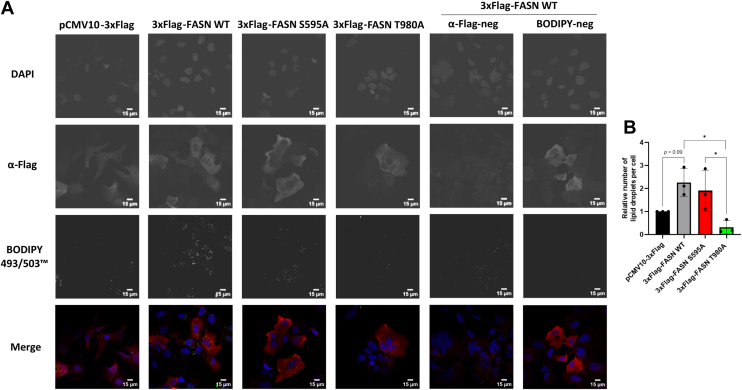
**FASN T980 is crucial for FASN activity**. Hep3B cells were transfected with an empty vector or 3xFlag-FASN (wild type and mutants) expressing vectors and incubated with the lipid droplet marker BODIPY 493/503 (n = 3) (*A*). Lipid droplets were counted using the ImageJ software (*B*). Confocal microscopy analysis indicates that FASN T980A mutation significantly impacts lipid droplets formation (bar size: 15 μm). Data are presented with means ± SD. ∗*p* < 0.05.

Since T980 *O*-GlcNAcylation site seems essential for FASN subcellular localization and dimerization, we then investigated its enzymatic activity by labeling lipid droplets (LDs) with the green fluorescent dye BODIPY 493/503 in Hep3B cells transfected with FASN expressing vectors (red staining) (Fig. 4*A*). LDs were then counted (Fig. 4*B*). When wild type FASN was overexpressed, LDs were increased compared to control cells (transfected with empty vector). However, when the T980A FASN-encoding vector was transfected, Hep3B cells displayed a significantly reduced number of LDs compared to wild type and FASN S595A, suggesting that *O*-GlcNAcylation at T980 is required for FASN activity.

### The FASN T980 O-GlcNAcylation site is crucial for cell survival, proliferation, and cell cycle progression of Hep3B cells

**Figure 5:**
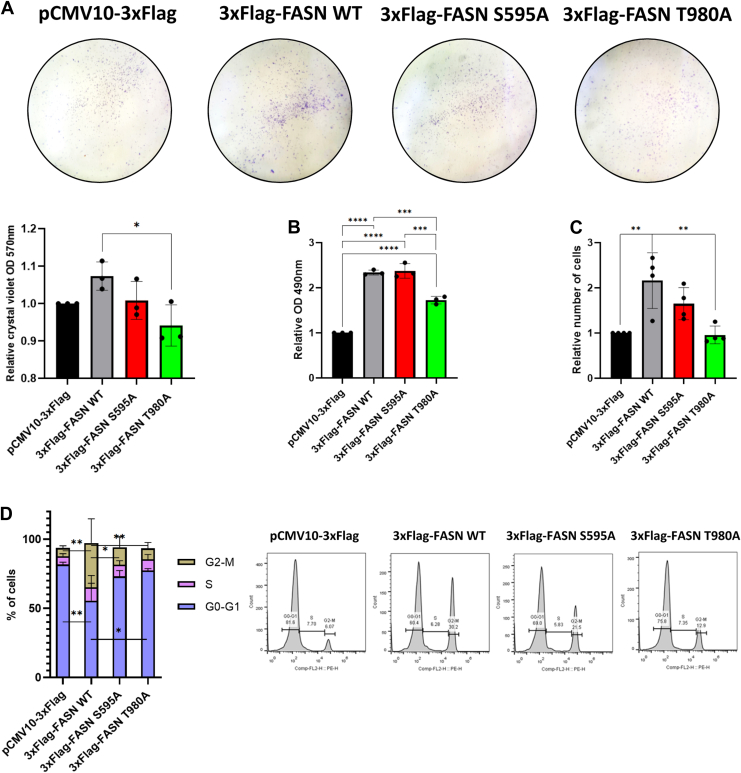
**FASN *O*-GlcNAcylation at T980 is pivotal for Hep3B cells survival, proliferation, and cell cycle progression.** Low-density Hep3B cells were transfected with an empty vector or 3xFlag-FASN (wild type and mutants) expressing vectors. Six days later, colonies were fixed and stained with crystal violet (n = 3) (*A*). Using MTS assay (*B*), cell survival was evaluated according to densitometry measured at λ = 490 nm (n = 6). Cell proliferation was determined by cell counting (n = 4) (*C*). Cell cycle was analyzed by flow cytometry (n = 3) (*D*). Data are presented with means ± SD. *∗p < 0.05; ∗∗p* < 0.01; ∗∗∗*p* < 0.001; ∗∗∗∗*p* < 0.0001.

FASN is involved in different hallmarks of cancer, which strongly emphasizes its pro-oncogenic nature (8). Thus, we next focused on the involvement of S595 and T980 *O*-GlcNAcylation sites on FASN's ability to promote Hep3B cells' survival, proliferation, and cell cycle progression. Using clonogenicity assays (Fig. 5*A*), we observed an increase in colony formation for cells overexpressing wild-type FASN compared to empty vector-transfected cells. Although no noticeable difference was observed for the S595A mutant, an apparent decrease in cell survival occurred for the T980A mutant. MTS assays were performed to validate these observations and showed significant differences for the T980A mutant compared to the wild type FASN (Fig. 5*B*). Then, cell counting, 24 hours after transfection, highlighted the ability for wild-type and S595A FASN isoforms to increase cell proliferation in contrast to the T980A mutant (Fig. 5*C*). At last, flow cytometry analysis showed a significant increase in cells in G2/M phase following overexpression of wild-type FASN (and of S595A mutant to a lesser extent), while the T980A mutant exhibited a profile similar to empty vector (Fig. 5*D*). Taken together, our data strongly suggest that FASN *O*-GlcNAcylation at T980 is essential to sustain Hep3B cell survival, proliferation, and cell cycle progression.

## Discussion

FASN is a major pro-oncogenic enzyme in the liver as its expression can play a crucial role in the initiation and progression of hepatic tumorigenesis (19). Increased *de novo* lipogenesis, promoted by the Warburg effect, is a recurrent cancer hallmark with FASN as the pivotal enzyme involved in deregulating this metabolism (8). Thus, investigation of FASN regulation in HCC is of particular importance. Here, we show that the nutrient-dependent modification *O*-GlcNAcylation positively regulates fatty acids synthesis. This observation correlates with previous reports showing that *O*-GlcNAcylation promotes FASN expression and stability in mice livers, HepG2 cells (9, 15), and HeLa cells (17). Due to its pivotal position in cell homeostasis, we wondered which *O*-GlcNAc sites of FASN could be involved in this positive regulation.

**Figure 6:**
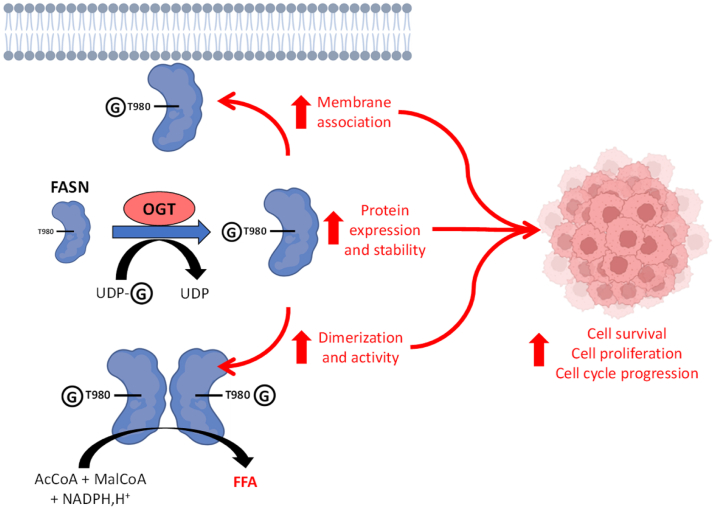
**FASN T980 is crucial for various properties of Hep3B cells.** The *O*-GlcNAcylation at T980 is crucial for FASN expression, stability, membrane residence, homodimerization, and activity, promoting Hep3B cells survival, proliferation, and cell cycle progression. This highlights the *O*-GlcNAcylation of FASN at T980 as a potential key modification that supports hepatic carcinogenesis.

Thus, we identified FASN S595 and T980 residues as major *O*-GlcNAcylation sites. T980 was already reported by Woo and collaborators (18). Protein sequences alignment indicated that S595 is well conserved among various species, with some discrepancies for T980 suggesting that this site has a major influence on lipogenic flow from one species to another (Fig. S5). Indeed, mutation of the two sites demonstrated that T980 has a key role in the behavior of FASN, notably altering the ability of the enzyme to dimerize, limiting the formation of lipid droplets (Fig. 6). However, mutation at T980 does not impact FASN folding as shown by prediction done with AlphaFold3 (Fig. S6). Since it has been reported that a pool of FASN protein is associated with lipid microdomains in prostate cancer cells (6), our observation also suggests that mutation at T980 reduces this interaction. While we did not look at the partitioning of the enzyme in either its wild-type form or mutated form, we showed that a mutation at T980 might reduce the interaction of FASN with membrane components. Further experiments are necessary to determine whether the reduction of FASN localization at the cell membrane is associated with decreased residence in lipid microdomains.

FASN activity is increased in tumor cells, and an elevation of *O*-GlcNAcylation is usually observed during carcinogenesis; we thus suggested that *O*-GlcNAcylation at T980 can be required to assist the pro-oncogenic features of FASN. We therefore evaluated the incidence of mutations at S595 and T980 on cell survival, proliferation, and cell cycle progression. As expected, mutation at T980 disturbed these processes when compared to the wild-type enzyme, but further investigations are nevertheless necessary to check this hypothesis.

However, our observations are strengthened by a recent study by Köberlin and collaborators (20). Indeed, while the authors highlighted an anticipatory lipid checkpoint in G1-phase mediated by FASN, our flow cytometry experiments (Fig. 5*D*) also support the ability of FASN to promote cell cycle progression.

Owing to the role of nutrient-sensing of *O*-GlcNAcylation, our findings align with previous reports highlighting FASN *O*-GlcNAcylation as a modification that promotes cancer cell survival during starvation (17). Similar to key mutations in oncoproteins (G12 in KRAS, V600 in BRAF, or T41 in β-catenin), we investigated whether mutations at T980 occur in FASN across various cancers. To our knowledge, no hotspot has been reported for FASN in cancer. Nevertheless, due to the close relationship between *O*-GlcNAcylation processes and nutritional status (the donor of the GlcNAc group being UDP-GlcNAc positioned at the intersection of different metabolisms), it will be interesting to evaluate in the near future the activity of FASN in pathologies associated with nutritional disorders or for which increased OGT activity has been reported.

The FASN interactome could also be a focus of attention to providing valuable insights, advancing our understanding of the functional role of T980 site-specific function. Deng and collaborators recently identified Heat shock protein 90 α (Hsp90α) as a crucial partner of FASN, enhancing its stability in HCC (21). While it is known that FASN *O*-GlcNAcylation plays a role in promoting its expression and activity, our work sheds additional light by identifying the T980 residue as a promising pivotal element for the etiology of HCC. It can be hypothesized that modification of FASN by OGT at T980 provides the protein with an interacting surface enhancing Hsp90α binding to the enzyme, resulting in increased stability and, consequently, activity.

Taken together, our results pave the way for future exploration of targeting FASN at T980, offering potential therapeutic avenues for patients with hepatic cancer.

## Experimental procedures

### Reagents, siRNA, and antibodies

C75, MG132 (MedChemExpress), and Thiamet-G (generously provided by Pr. David Vocadlo, Simon Fraser University, Burnaby, CA) were dissolved in dimethyl sulfoxide (DMSO) at a concentration of 100 mM, 50 mM and 1 mM as stock solutions, respectively. Control siRNA (si*CTRL#1* - MISSION siRNA universal negative control) and siRNA targeting human *OGT* (si*OGT#1*: GGAGGCUAUUCGAAUCAGU[dT][dT] forward, ACUGAUUCGAAUAGCCUCC[dT][dT] reverse) were purchased from Sigma-Aldrich or from Dharmacon for si*CTRL*#2 (ON-TARGETplus Non-targeting Pool), si*OGT#2* (ON-TARGETplus Human OGT (8473) siRNA), and si*OGA* (siGENOME Human MGEA5 (10,724) siRNA). The antibodies used are listed in Table S1.

### Cell culture

The human hepatoblastoma HepG2 and hepatocarcinoma Hep3B cell lines were respectively provided by the EGID (European Genomic Institute for Diabetes) and ONCOLille institutes in Lille. HepG2 and Hep3B cell lines are ATCC certified. Both cell lines, tested and free from *mycoplasma*, were cultured in Minimal Essential Medium (MEM, Biowest) supplemented with 2 mM L-glutamine, 1 mM sodium pyruvate, 10% (v/v) fetal calf serum (FCS) (Dominique Dutscher), and penicillin/streptomycin at 1:100 dilution (Gibco). Cells were incubated at 37 °C, in a 5% (v/v) CO_2_-enriched, humidified atmosphere.

### Cell treatment and cell transfection

HepG2 cells were treated 24 h with C75 or Thiamet-G at a final concentration of 100 μM and 1 μM, respectively, in cell culture medium. DMSO was used as a negative control (vehicle). For RNA silencing, HepG2 cells were reverse-transfected with small interfering RNA (siRNA)—si*CTRL#1/#2*, si*OGT#1/#2* or si*OGA* at 10 nM—using Lipofectamine RNAiMAX (Invitrogen) as described in (22). Ninety-six hours later, cells were harvested in Phosphate-Buffered Saline (PBS).

Hep3B cells were transiently transfected when 70% of confluence was reached for 24 h with empty vector (negative control) or plasmids encoding 3xFlag-FASN (wild type or mutant isoforms; 1 μg/well in 6-well plate) using jetOPTIMUS according to the manufacturer's instructions (Polyplus).

### Cycloheximide chase assay

CHX chase assays were performed to assess FASN stability. Cells were seeded in 6-well plates and grown to ∼70% confluence. 24 h after transfection, protein synthesis was inhibited by treating cells with 40 μg/ml CHX (Sigma) for the indicated time periods.

### Flow cytometry

Cell cycle progression of Hep3B transfected cells was monitored by propidium iodide (PI) staining and analysis by flow cytometry, as previously described (22).

### Cell proliferation assay

Hep3B cells transfected with 3xFlag-FASN for 24 h in T25 flasks were washed with PBS and harvested by trypsinization. After trypsin neutralization by complete medium addition, cells were collected and a fraction was mixed (50:50) with 0.04% (v/v) trypan blue in PBS for cell counting using an automatic cell counter.

### Cell viability assays

For MTS assays, 1000 Hep3B cells were seeded in 96-well plates and transfected with 3xFlag-FASN for 24 h. Cell viability was then measured using the MTS reagent (Promega) according to the manufacturer's instructions.

For clonogenicity assays, 8000 Hep3B cells were seeded in 6-well plates and transfected with 3xFlag-FASN for 24 h, followed by a medium change. Six days after transfection, cells were washed with ice-cold PBS before fixation with 4% (w/v) paraformaldehyde (PAF) in PBS at room temperature (RT) for 30 min. After three washes, surviving colonies were stained with 0.05% (v/v) Crystal violet in MilliQ water for 15 min with slight shaking and in the dark. Cells were finally washed with MilliQ water and colonies counted with ImageJ.

### Immunofluorescence and confocal microscopy

Hep3B cells transfected with 3xFlag-FASN on coverslips were incubated for 1 h with BODIPY (493/503) (Invitrogen) diluted at 1:1000 in the cell culture medium. Then cells were washed in ice-cold PBS before fixation with 4% (w/v) PAF in PBS at RT for 30 min. After three washes, cells were permeabilized with 0.5% (v/v) Triton X-100 in PBS for 20 min. After three new washes, coverslips were incubated with blocking buffer (2% (v/v) FCS, 2% (w/v) bovine serum albumin and 0.2% (w/v) gelatin in PBS) for 1 h at RT and subsequently incubated with the primary antibodies (α-Flag M2, 1:100) diluted in the blocking buffer, overnight at 4 °C. Then coverslips were washed three times with PBS and incubated with Alexa Fluor 546-conjugated secondary antibodies (1:600) diluted in blocking buffer (Table S1) for 1 h in the dark, at RT. After three new washes, nuclei were labeled with DAPI solution at 1 μg/ml for 10 min at RT. The coverslips were rinsed with ultrapure water and mounted on slides using Mowiol medium (Calbiochem, Merck chemicals). Negative controls were done using only the secondary antibodies. The fluorescence was detected with an inverted Zeiss LSM780 confocal microscope with a 63 × oil immersion lens at room temperature and data were collected with the ZEN software (Zeiss, Oberkochen, Germany). Images were analyzed with ImageJ.

### Cell fractionation

Transfected Hep3B cells in T25 flasks were fractionated to separate membrane and cytosolic fractions using the commercial Subcellular Proteome Extraction Kit (Merck) according to the manufacturer's instructions.

### Cell lysis, SDS-PAGE, and Western blotting

Cells were first washed with ice-cold PBS and then incubated for 10 min with lysis buffer (50 mM Tris–HCl, 150 mM NaCl, 0.1% (w/v) sodium dodecyl sulfate, 1% (v/v) Triton-X100, 0.25% (w/v) sodium deoxycholate, pH 7.5) containing protease inhibitors (protease cocktail inhibitors, Sigma-Aldrich), 50 mM sodium fluoride (Sigma-Aldrich) and 1 mM sodium orthovanadate (Sigma-Aldrich). The cell lysates were then centrifuged at 20,000*g* for 15 min at 4 °C. The supernatants were collected and protein concentrations were measured using the micro BCA protein assay kit (Thermo Fisher Scientifc) according to the manufacturer's instructions. Proteins were mixed with 5X Laemmli buffer (250 mM Tris-HCl, 5% (w/v) SDS, 5% (v/v) β-mercaptoethanol, 40% (v/v) glycerol, pH 6,8) and heated at 95 °C for 7 min. Then, proteins (20 μg per lane) were resolved by 8% SDS-PAGE in electrophoresis buffer (25 mM Tris–HCl, 192 mM glycine, 0.1% (w/v) SDS, pH 8.8) and transferred onto nitrocellulose membranes (HybondTM-C EXTRA, GE Healthcare) in transfer buffer (25 mM Tris–HCl, 192 mM glycine, 20% (v/v) methanol, pH 8.8). Membranes were stained with a Ponceau red solution (5% (v/v) acetic acid and 0.1% (w/v) Ponceau red) to assess equal loading. Membranes were destained with TBS (Tris-Buffered Saline) - Tween20 (20 mM Tris–HCl, 150 mM NaCl, 0.05% (v/v) Tween20 (Sigma-Aldrich), pH 7.5) (TBS-T), subsequently blocked in 5% (w/v) nonfat dry milk in TBS-T, and probed overnight at 4 °C with primary antibodies (Table S1). After three TBS-T washes, membranes were incubated with the appropriate horseradish peroxidase-conjugated secondary antibody (anti-mouse or anti-rabbit IgG-HRP linked, Table S1) for 1 h at RT. After three TBS-T washes, blots were revealed using enhanced chemiluminescence (West Pico Plus or Femto, ThermoScientifc). The images were acquired using a CCD camera (Fusion Solo, Vilbert Lourmat). For additional probing, the membranes were stripped with the antibody stripping solution (Gene Bio-Application L.T.D.) for 15 min at RT, then washed in TBS-T and re-probed with primary antibodies. Densitometry analyses of Western blot images were done using Image J software.

### Native-PAGE

Native-PAGE followed the same steps as the *Western blotting* section, but with the following modifications. Cells were lysed in 10 mM Tris/HCl, 1 mM EDTA, 1 mM EGTA, 0.5% (v/v) Triton X-100 lysis buffer, protease inhibitors, pH 7.5. Proteins were mixed with loading buffer (20 mM Tris-HCl, 50% (v/v) glycerol, bromophenol blue, pH 8.8) and run on a 4-to-10% acrylamide/bisacrylamide gel gradient (for note, 25% (v/v) glycerol was added in the 10% acrylamide/bisacrylamide gel solution). Finally, the migration step was done in electrophoresis buffer (25 mM Tris-HCl, 192 mM glycine, 0.1% (w/v) SDS, pH 8.8) at 50 V overnight at 4 °C.

### Immunoprecipitation and mass spectrometry analysis

Data were generated from four individual experiments. HepG2 cells were lysed in lysis buffer as in the *W. blotting* section. For the pre-clearing step, 700 μg of proteins were incubated with 20 μl of agarose-beads coupled to A/G proteins (50:50) (Cytiva) for 1 h at 4 °C. After centrifugation (2 min, 200 g, 4 °C), the supernatant was incubated overnight with 5 μg of anti-FASN antibody (Abcam, rabbit polyclonal, ab99359) or 5 μg of rabbit IgG-UNLB (Southern Biotech) for negative control. Then, 40 μl and 20 μl of agarose-beads coupled to A and G proteins were respectively added to samples and incubated 1 h at 4 °C. After three washes with lysis buffer, 25 μl of Laemmli 5X buffer were added and samples were heated 7 min at 95 °C. Proteins were resolved by 8% SDS-PAGE. The gel was stained with commercial Quick Coomassie Stain (NeoBiotech) according to the manufacturer's instructions.

The protein band of interest was digested with trypsin based on the protocol described by Helle *et al.* (23). The peptides were then analyzed by LC-MS/MS using an Orbitrap Q-exactive plus. Briefly, 1 μl was injected with solvent A (5% (v/v) acetonitrile and 0.1% (v/v) formic acid) for 3 min at a flow rate of 5 μl.min^−1^ on an Acclaim PepMap100 C18 pre-column (5 μm, 300 μm i.d. × 5 mm) from ThermoFisher Scientific. The peptides were then separated on a C18 Acclaim PepMap100 C18 reversed phase column (3 μm, 75 μm i.d. × 500 mm), using a linear gradient (1–40%) of solution B (75% (v/v) acetonitrile and 0.1% (v/v) formic acid) at a rate of 250 nl.min^-1^ during 60 min. The column and the pre-column were placed in an oven at a temperature of 45 °C. The LC runs were acquired in positive ion mode with MS scans from *m/z* 350 to 1500 in the Orbitrap mass analyzer at 70,000 resolution at *m/z* 200. The automatic gain control was set at 1e6, and the maximum ion time was set to 90 ms. Sequentially, MS/MS scans were acquired in the high-energy collision dissociation cell for the 15 most intense ions detected in the full MS survey scan. Automatic gain control was set at 5e105, and the normalized collision energy was set to 28 eV. The resolution was set at 17,500 at *m/z* 200. The automatic gain control was set at 5e5, and the maximum ion time was set as 140 ms. The isolation windows for MSMS were set at 2 *m/z*. Dynamic exclusion was set at 90 s, and ions with 1 or more than 8 charges were excluded.

The acquired raw files were analyzed with Proteome Discoverer 2.4 (ThermoScientific) using a custom-made database including FASN sequence. The peptide mass tolerance was set to 10 ppm and 0.05 Da for MS/MS. Variable modifications included were as follows: oxidation of Met and Pro, deamidation of Asn and Gln, phosphorylation of Tyr, Ser, and Thr, pyro-Glu, and *O*-GlcNAcylation of Ser and Thr. Fixed modifications included carbamidomethylation of Cys. For high-confidence, the peptide threshold was FDR 1% and proteins were identified with two peptides (24).

### Co-immunoprecipitation

Hep3B cells were lysed in co-IP buffer (50 mM Tris/HCl, 150 mM NaCl, 0.5% NP-40 (v/v), and protease inhibitors, pH 8.0). 20 μg of proteins were set aside for control (input). For the pre-clearing step, 500 μg of proteins were incubated with 50 μl of agarose-beads coupled to A/G proteins (50:50) (Cytiva) for 1 h at 4 °C. After centrifugation (3 min, 300 g, 4 °C), the supernatant was incubated overnight with 2.5 μg of DM17 antibody or 2.5 μg of rabbit IgG-UNLB (Southern Biotech) for negative control. Then, 20 μl and 10 μl of agarose-beads coupled to A and G proteins were, respectively, added to samples and incubated 1 h at 4 °C. After three washes with co-IP buffer, 20 μl of Laemmli 5X buffer were added and samples were heated 7 min at 95 °C. Proteins were resolved by 8% SDS-PAGE followed by Western blotting.

### WGA-beads enrichment

Protein samples from Hep3B-transfected cells were diluted into co-IP buffer until reaching a final dilution of 500 μg of protein per mL. Then, samples were incubated with 20 μl of WGA-agarose beads (VectorLabs) with or without 0.5 M GlcNAc overnight at 4 °C to check specificity of WGA. WGA-bound proteins were collected, washed four times with washing buffer (10 mM Tris/HCl, 100 mM NaCl, 0.4% (w/v) sodium deoxycholate, 0.3% (w/v) SDS, and 0.2% (v/v) NP-40, pH 7.5), resuspended in Laemmli buffer, boiled, resolved by SDS-PAGE, and analyzed by Western blot.

### Fatty acids analysis by GC-FID

After treatment with Thiamet-G or C75, or siRNA transfection, HepG2 cells were scraped in PBS and lyophilized in a Teflon-lined screw-capped glass tube. Then, 1 ml of freshly prepared 5% H_2_SO_4_ (v/v) in methanol was added and completed with 300 μl of toluene. The mix was vortexed vigorously for 30 s before heating for 1 h at 90 °C. Fatty acid methyl esters (FAMES) were extracted with 1.5 ml of 0.9% (w/v) NaCl and 1 ml of heptane. The tube was vortexed and then centrifuged briefly to facilitate phase separation. The heptane extracts (upper organic phase) were transferred to a new glass tube and evaporated under a stream of N_2_, then dissolved in 50 μl heptane. FAME composition was determined by Gas phase Chromatography with a downstream Flame Ionization Detector (GC-FID). The GC-FID analyses were carried out on a Thermo Scientific Trace GC Ultra chromatography, and chromatographic separation was performed on a BPX70 column (30 m × 0.25 mm id, film thickness 0.25 μm) with helium as carrier gas and according to the following program: 150 °C for 3 min, then temperature gradient from 150 °C to 240 °C at a rate of 10 °C/min, and finally 240 °C for 5 min. One μl of sample was injected in a 260 °C inlet with a 40:1 split ratio. Methyl ester derivatives were detected using a FID at 260 °C.

### FASN cloning

To clone the FASN gene into the pCMV10-3xFlag expression vector, the Hind III - FASN sense (5' → 3') and FASN - Xba I antisense (3' → 5') primers (Eurogentec) (Table S2) were used to amplify the FASN sequence by PCR from the vCMVp-mCherry-FASN-V5Tag vector (eZyvec). After preparing the reaction mixture (1X GC buffer, 3% (v/v) DMSO, deoxyNucleotide TriPhosphates at 200 μM each), sense and antisense primers (0.5 μM), Phusion polymerase (0.02 U/μl; NEB) and 25 ng of vCMVp-mCherry-FASN-V5Tag plasmid), PCR was carried out under the following conditions: 2 min at 95 °C followed by 30 cycles of denaturation (30 s, 95 °C)—primer hybridization (1 min, 60 °C)—elongation (4 min, 72 °C) and 10 min at 72 °C. The amplicons and pCMV10-3xFlag vector were then incubated with Hind III and Xba I restriction enzymes (NEB) to generate cohesive ends at the insert and vector level. The addition of T4 DNA ligase enabled ligation.

### Site-directed mutagenesis by PCR

Site-directed mutagenesis was performed using the QuikChange II XL Site-Directed Mutagenesis Kit (Agilent). The mix used for the PCRs was as follows: 10X buffer (5 μl), vector (20 ng), sense and antisense primers (2.5 μl; 5 μM) (Table S2), dNTPs (1 μl), Quick Solution (3 μl), Pfu turbo ultra (1 μl; 2.5 U/μl), *qs* 50 μl Milli Q water. PCR conditions were carried out as follows: 60 s at 95 °C followed by 18 cycles of denaturation (95 °C, 50 s)—hybridization (60 °C, 50 s)—elongation (68 °C, 14 min) followed by 7 min at 68 °C. After PCR, the unmutated parent vector was digested by incubating the PCR mix with the kit's Dpn I enzyme (1 μl) for 1 h at 37 °C after centrifugation for a few seconds.

### Bacteria transformation

XL10 Gold or DH5α bacteria (*Escherichia coli*) were transformed with plasmids by incubating 45 μl of bacteria with 2 μl of β-mercaptoethanol and incubating for 10 min on ice. Next, 2 μl of PCR product was added before incubation for 30 min on ice followed by 30 s at 42 °C and then 2 min on ice. Bacteria were then incubated for 1 h at 37 °C in 500 μl of LB (Lysogeny Broth) medium with constant agitation before being plated on an LB agar with Carbenicillin (50 μg/ml final) Petri dish.

### Plasmid extraction and amplification

Plasmids were extracted from bacteria using the Plasmid DNA purification—nucleospin Plasmid kit (Macherey Nagel). A colony was seeded in 5 ml LB (+antibiotic) at 37 °C overnight. The following day, 450 μl of bacteria were mixed with 50 μl of DMSO for storage at −80 °C. The remaining bacteria were centrifuged at 2,400*g* for 15 min at room temperature and plasmids extracted according to the manufacturer's instructions. DNA amounts were assayed with NanoDrop by measuring absorbance at 260 nm, and plasmids were sequenced by Eurofins Genomics. Plasmids were amplified using the NucleoBond Xtra-Midi kit (Macherey Nagel) according to the manufacturer's instructions. DNA amounts were assayed with NanoDrop.

### FASN dimer structure prediction

All FASN dimer structures were predicted with AlphaFold3 (25) with default parameters. ipTM and pTM scores are respectively 0.68 and 0.7 for the wild type structure, 0.67 and 0.69 for T980A and 0.67 and 0.69 for *O*-GlcNAcylated T980. The respective Predicted Aligned Error matrices are provided below each structure in Fig. S6. The rendering and Root Mean Square Deviation computations were performed with PyMol 2.3.0 (The PyMOL Molecular Graphics System, Version 2.3.0 Schrödinger, LLC.).

### Statistical analysis

Data are represented with means ± SD and were compared using one-way or two-way ANOVA and Student's *t* test (two-tailed and unpaired *t* test). Statistical analyses were performed using Graph-Pad Prism 8.0.2 (GraphPad Software, Inc.) software.

## Data availability

All data generated or analyzed during this study are included in this published article and its supplementary information files.

## Supporting information

This article contains supporting information.

## Conflict of interest

The authors declare that they have no conflicts of interest with the contents of this article.
