# Supplementary material for: O-GlcNAcylation of fatty acid synthase is required for its proper subcellular localization, expression level, and activity
Source: J Biol Chem. 2025 Jul 18;301(8):110497. doi: 10.1016/j.jbc.2025.110497 (PMC12362114; doi:10.1016/j.jbc.2025.110497)
Supplement: Table S1 [file mmc1.docx]

**Supplementary table 1: Antibodies used in this study.**

| **Antibodies** | **Dilution for WB** | **Dilution for IF** |
| --- | --- | --- |
| **Primary antibodies (supplier, reference)** | | |
| Mouse monoclonal anti-*O*-GlcNAc (Thermo Scientific, RL2) | 1 : 1,000 |  |
| Rabbit polyclonal anti-OGT (Sigma-Aldrich, DM-17) | 1 : 1,000 |  |
| Mouse monoclonal anti-Flag M2 (Sigma-Aldrich, F1804) | 1 : 1,000 | 1 : 100 |
| Mouse monoclonal anti-β-actin (Merck, A1978) | 1 : 5,000 |  |
| Mouse monoclonal anti-GAPDH (Santa Cruz, 6C5) | 1 : 1,000 |  |
| Mouse monoclonal anti-E-Cadherin (Santa Cruz, G-10) | 1 : 1,000 |  |
| Rabbit monoclonal anti-OGA (Abcam, ab124807) | 1 : 10,000 |  |
| Rabbit polyclonal anti-Ubiquitin (Enzo Life Sciences, ADI-SPA-200) | 1 : 5,000 |  |
| **Secondary antibodies (supplier)** | | |
| Sheep anti-mouse IgG/HRP conjugated (GE Healthcare) | 1 : 10,000 |  |
| Donkey anti-rabbit IgG/HRP conjugated (GE Healthcare) | 1 : 10,000 |  |
| Goat anti-mouse AlexaFluor546 (Invitrogen) |  | 1 : 600 |
