# Supplementary material for: O-GlcNAcylation of fatty acid synthase is required for its proper subcellular localization, expression level, and activity
Source: J Biol Chem. 2025 Jul 18;301(8):110497. doi: 10.1016/j.jbc.2025.110497 (PMC12362114; doi:10.1016/j.jbc.2025.110497)
Supplement: Table S2 [file mmc2.docx]

**Supplementary table 2 : Primers used in this study.**

| **Primer type** | **Name** | **Sequence (5’ → 3’)** |
| --- | --- | --- |
| **Cloning** | Hind III_*FASN*_Fw | CCCAAGCTTATGGAGGAGGTGGTGATTGCCGGC |
|  | *FASN*_Xba I_Rv | GCTCTAGATTACGTAGAATCGAGTCCGAGGAGAGGGTTAGGGATAG |
| **Mutagenesis** | *FASN* S595A_Fw | CGACGGCTGCCTGGCCCAGGAGGAGGC |
|  | *FASN* S595A_Rv | GCCTCCTCCTGGGCCAGGCAGCCGTCG |
|  | *FASN* T980A_Fw | CCCAACCCCGCGGAGCCCCTCTTCCTG |
|  | *FASN* T980A_Rv | CAGGAAGAGGGGCTCCGCGGGGTTGGG |
| **Sequencing** | *FASN*_Fw1 | CGCCACCATCCTGAACG |
|  | *FASN*_Fw2 | TGATGACATCGTCCATTC |
|  | *FASN*_Fw3 | CTTCCCCAACGGTTCAG |
|  | *FASN*_Fw4 | GCTGAAGATGGTGGTGC |
|  | *FASN*_Fw5 | CTTGGTGAACTGTCTCC |
|  | *FASN*_Fw6 | GTCGCTTCCTGGAAATTG |
|  | *FASN*_Fw7 | GTGGAGACGATGAGCAC |
|  | hGH_PA_Rv | CAGCTTGGTTCCCAATAG |
