# Supplementary material for: O-GlcNAcylation of fatty acid synthase is required for its proper subcellular localization, expression level, and activity
Source: J Biol Chem. 2025 Jul 18;301(8):110497. doi: 10.1016/j.jbc.2025.110497 (PMC12362114; doi:10.1016/j.jbc.2025.110497)
Supplement: Figures S1–S6 [file mmc3.pdf]

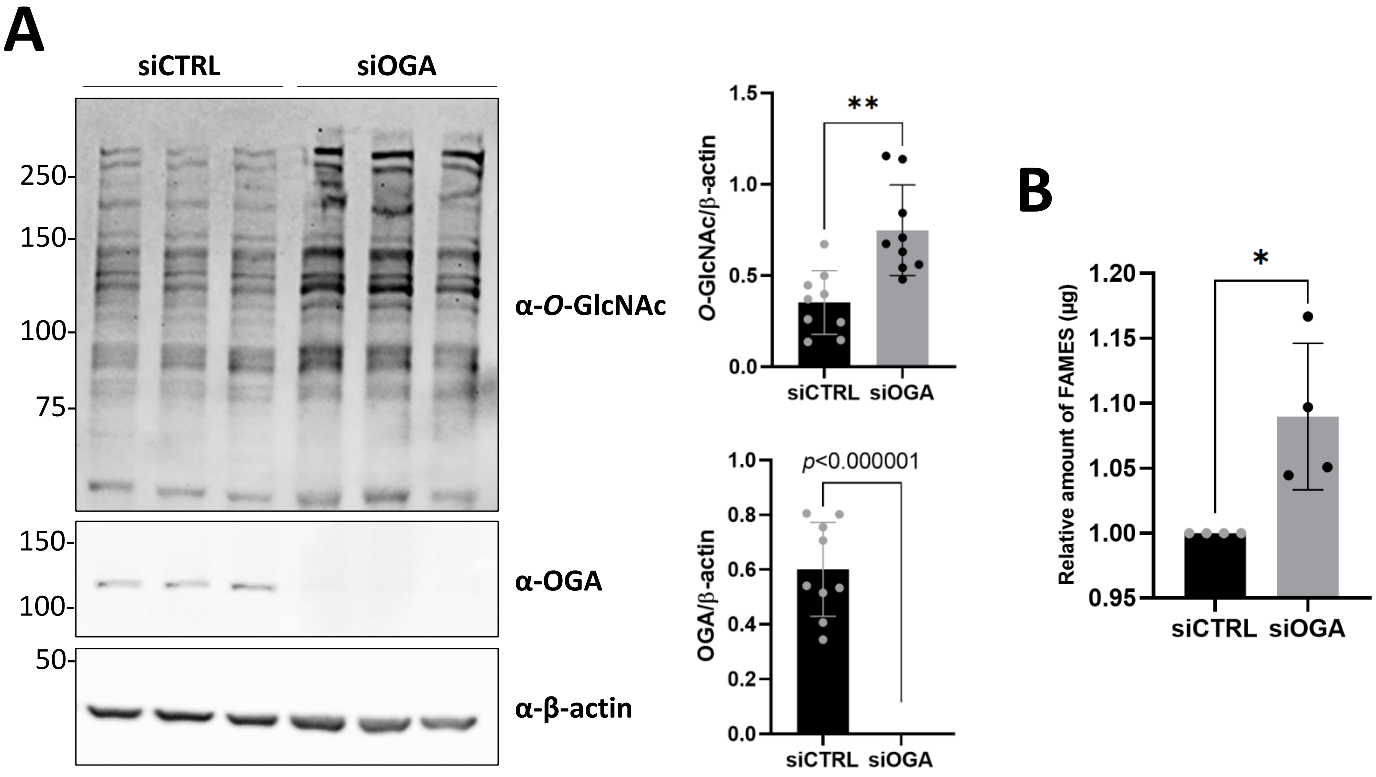

Supplementary Figure 1: OGA knockdown positively impacts fatty acids synthesis.

HepG2 cells were transfected with siRNA targeting OGA mRNA. Cell lysates were analyzed by Western blot (n=9) according to their OGA and *O*-GlcNAc contents (A). Molecular mass markers are indicated on the left (kDa). Optical densities were measured and normalized with β-actin expression. Global amounts of FAMES (B) from HepG2 transfected cells were measured by GC-FID (n=4). Data are presented with means ± SD. **p*<0.05; ***p*<0.01.

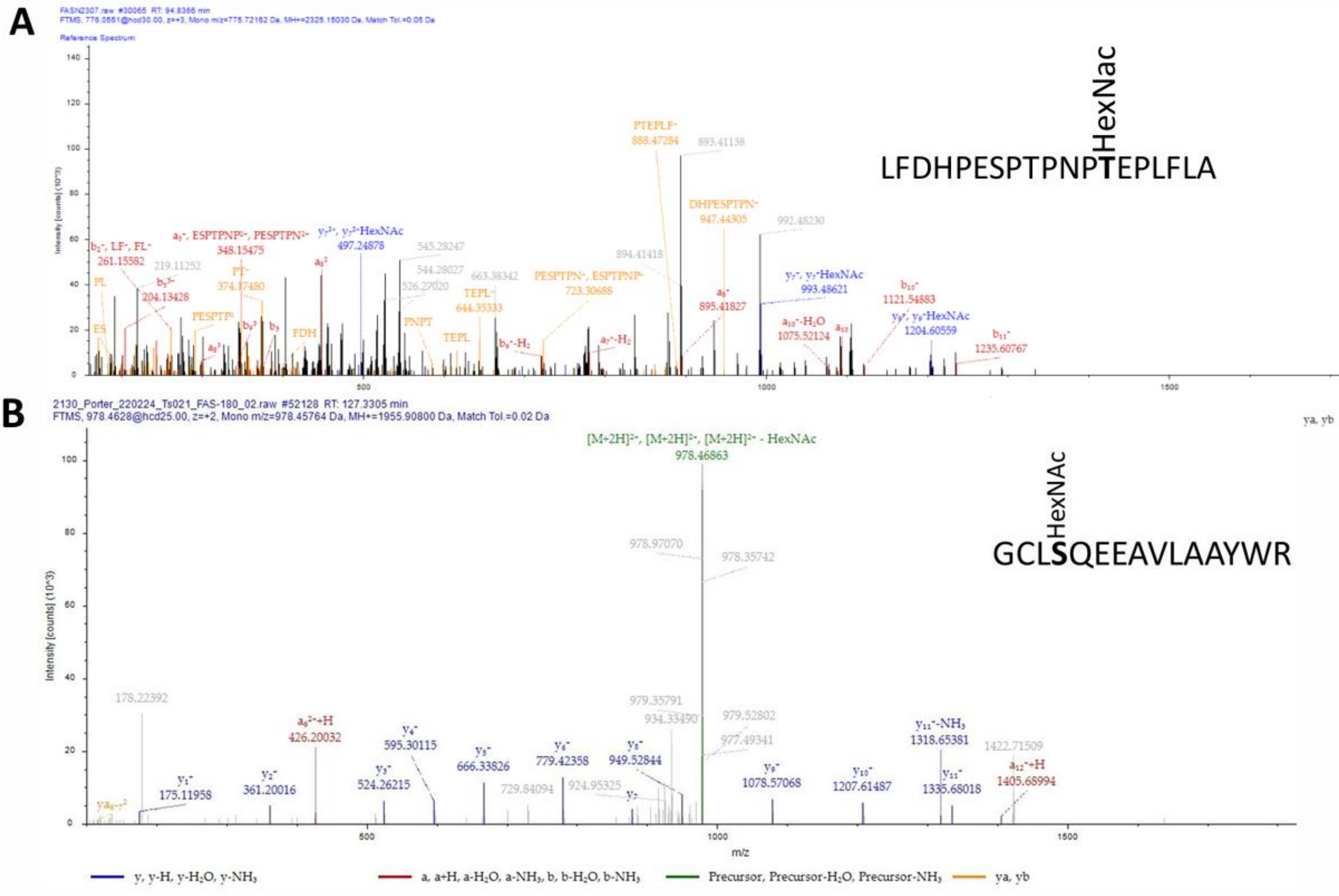

Supplementary Figure 2: FASN is *O*-GlcNAcylated at S595 and T980.

LC-MS/MS analyses of immunopurified FASN revealed threonine 980 (A) and serine 595 (B) as FASN *O*-GlcNAcylated sites.

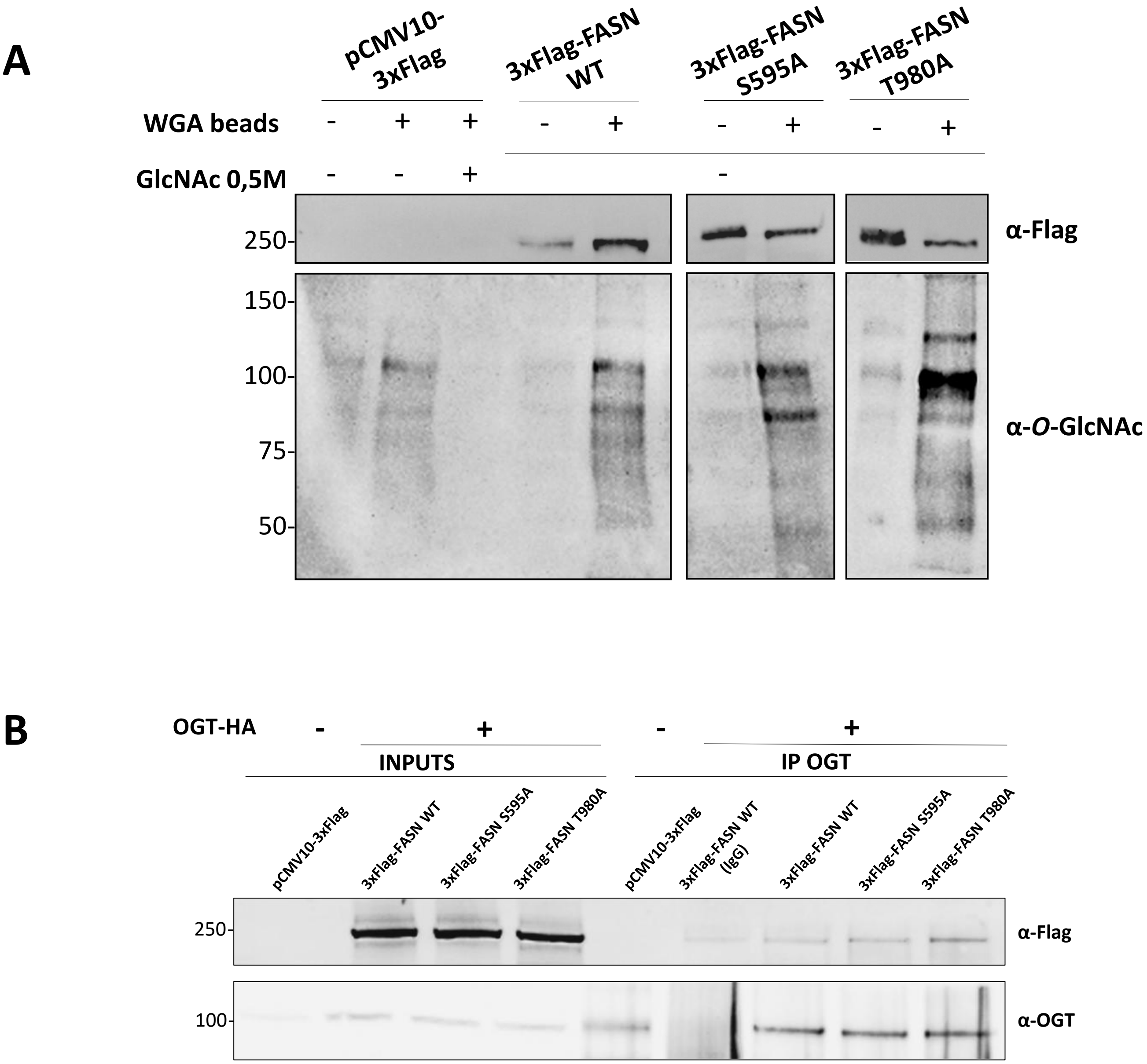

Supplementary Figure 3: Evaluation of wild type and mutant FASN forms glycosylation status and interaction with OGT.

The different forms of FASN were enriched on WGA-coupled beads. Bound proteins were resolved by SDS-PAGE, electroblotted onto nitrocellulose and probed with anti-Flag or anti-*O*-GlcNAc antibodies (**A**). Co-immunoprecipation of OGT-HA followed by anti-Flag (FASN) and anti-OGT immunoblotting was performed with the different forms of FASN (**B**).

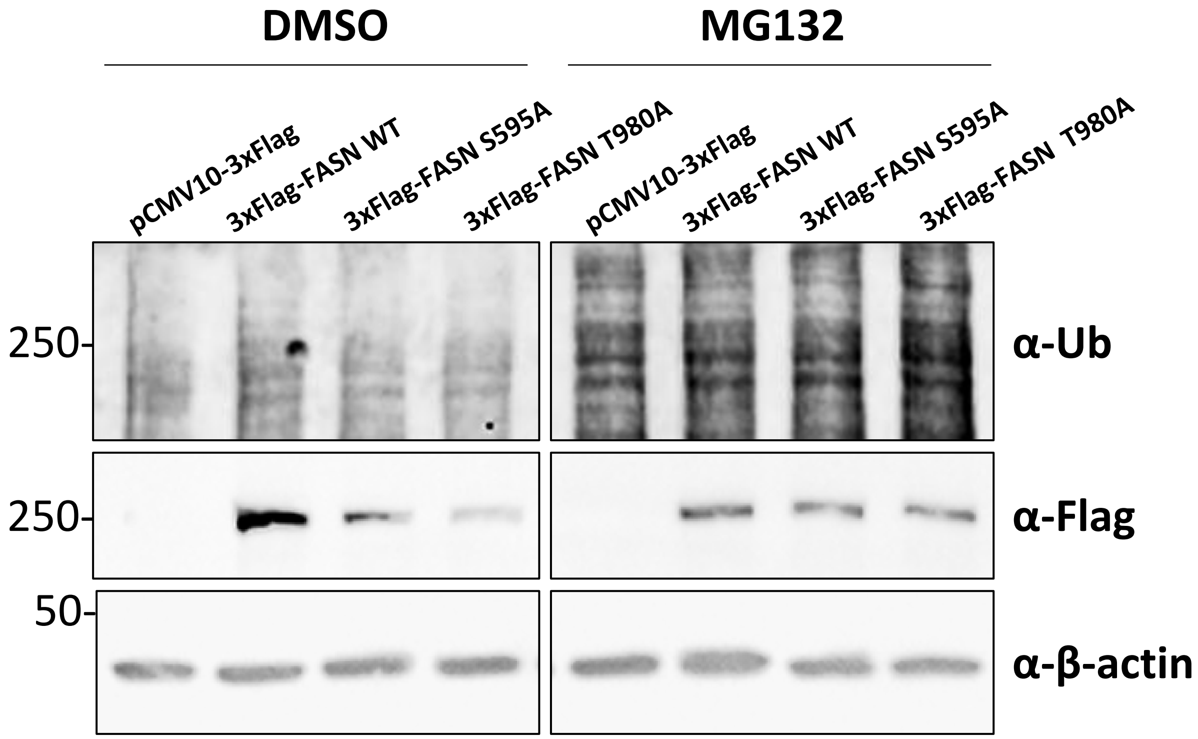

Supplementary Figure 4: Inhibiting proteasome restores in part expression of mutant FASN proteins.

Hep3B cells were transiently transfected with the various plasmids encoding either wild type or mutant FASN, or with an empty vector. Cells were then treated with the proteasome inhibitor MG132 (4h, 5 µM) and expression of the different forms of FASN was evaluated by Western blot. The efficiency of MG132 was verified by probing whole proteins with an anti-ubiquitin antibody.

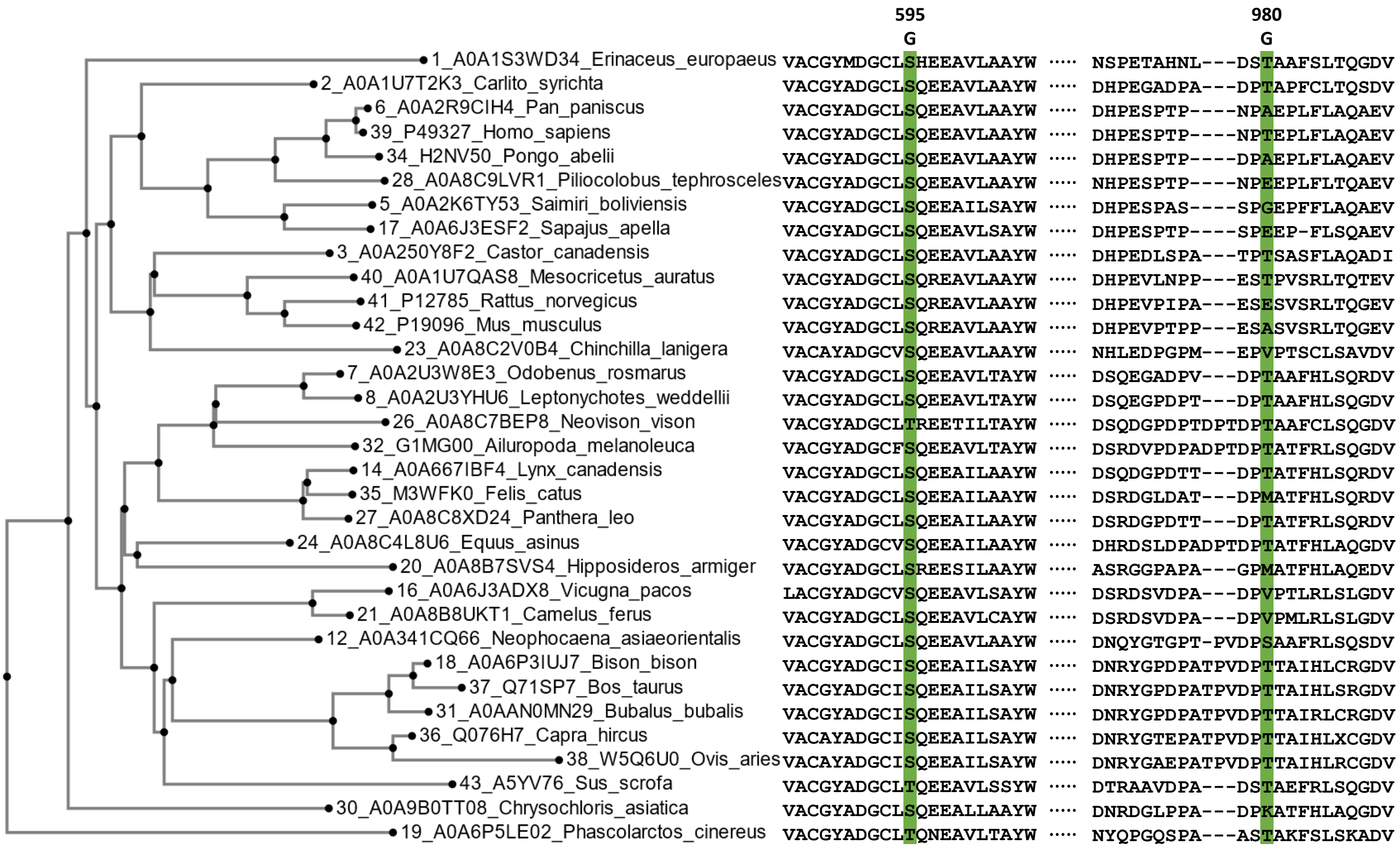

Supplementary Figure 5: Mammalian FASN protein sequence alignments.

Various species FASN protein sequences were retrieved from UniProt database and aligned using MAFFT V7 with default settings. Sequences with more than 97% similarity were removed for visualization purposes. S595 and T980 are conserved among mammals at 88% and 58%, respectively.

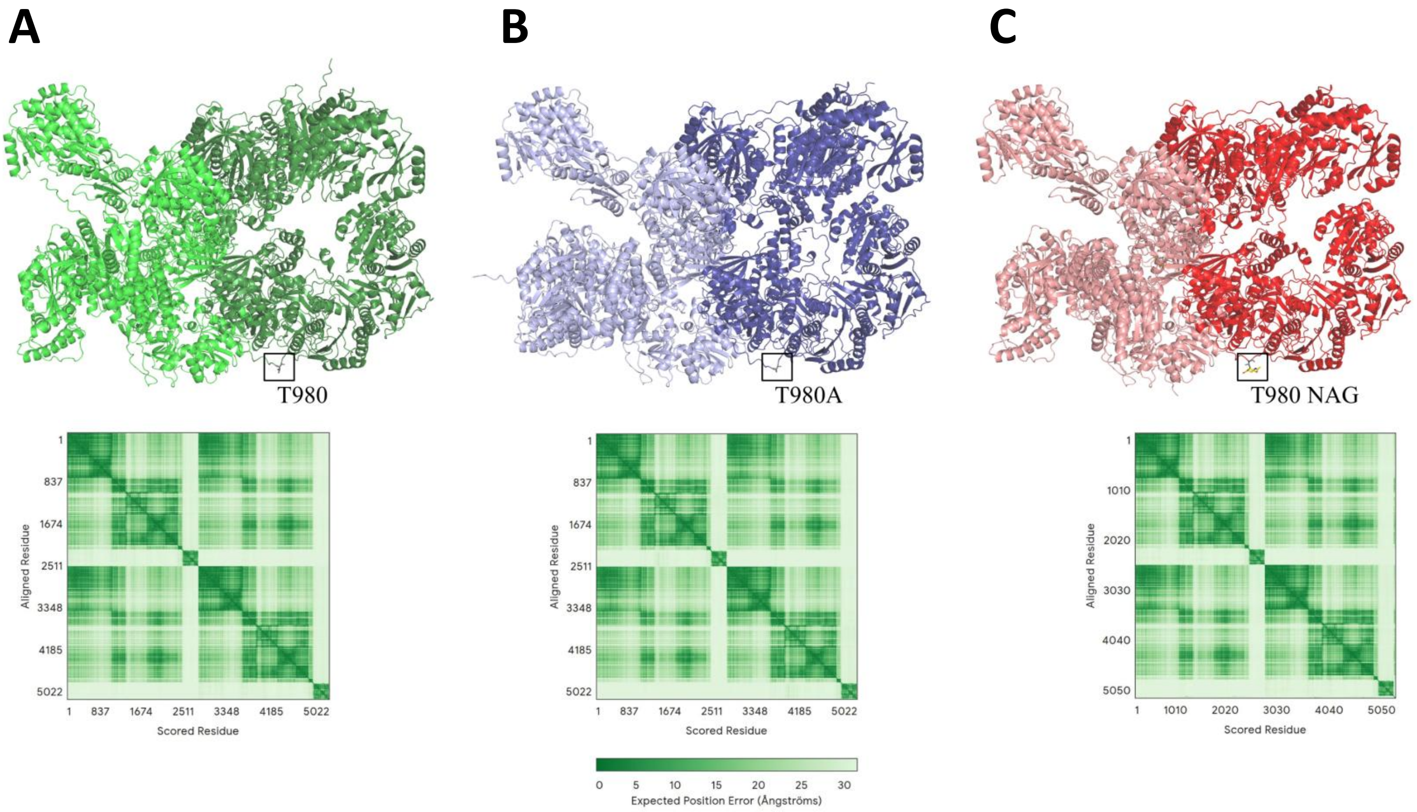

Supplementary Figure 6: FASN dimer structures.

FASN WT (**A**), T980A (**B**) and *O*-GlcNAcylated at T980 (**C**) dimer structures were predicted by the AlphaFold3 server; each dimer is coded in two shades of green (WT), blue (T980A) and red (*O*-GlcNAcylated at T980), one shade per chain, in cartoon representation; T980 and T980A are represented in grey in stick mode; the N-AcetylGlucosamine (NAG) is shown in yellow, in stick mode. Below each structure is depicted the corresponding Predicted Aligned Error matrix. The Root Mean Square Deviation (RMSD) calculated between T980A and WT is 1.911 Å; the RMSD between *O*-GlcNAcylated T980 and WT is 1.690 Å.
